# Americans’ ideological differences have decreased by race, increased by education

**DOI:** 10.1093/pnasnexus/pgag102

**Published:** 2026-04-03

**Authors:** Stephen Jessee

**Affiliations:** Department of Government, University of Texas, Austin, TX 78712, USA

**Keywords:** American politics, ideology, race, education

## Abstract

Racial differences in partisan vote have been studied extensively, with recent work documenting shifts in longstanding differences. Recently, attention has shifted to education as a dividing line in American politics. I analyze data from extremely large surveys in each of the last five presidential election years, focusing on the average ideological positions of racial and educational subgroups. I show that ideological differences have decreased sharply by race, driven primarily by Blacks becoming more conservative, but also Hispanics moving to the right. Over the same time period, differences in average ideology by education have grown dramatically, with less educated Americans being much more conservative and more educated Americans being much more liberal. These trends by education have occurred across different racial groups, but have been most dramatic among whites.

## Introduction

Commentary in popular media (1) as well as the academic literature (2, 3) has recognized race and ethnicity (hereafter shortened as “race”) as well as education as prominent political cleavages in American politics. In the past decade or two, partisan vote shares have shifted along both of these lines (4), with many arguing that the unique characteristics of Donald Trump were the primary driver of these changes (5). This recent discourse follows a much longer line of research exploring political divisions along such demographic characteristics (6).

A related but different question is how the political views of Americans divide across these demographic groupings, and how this has changed over time. To investigate this, I analyze a series of large-sample surveys fielded in presidential election years between 2008 and 2024 to construct policy-based ideology estimates for respondents that are comparable across years based on their views on specific issues. These estimates provide a measure of the liberal-conservative ideological positions of each respondent. The extremely large sample size of these surveys allows for the relatively precise estimation of the average ideologies among racial and educational subgroups.

Using these measures, I illustrate dramatic shifts in the average ideology of racial and educational subgroups in America. These results show that while ideological differences between the races have been decreasing over time, driven primarily by a shift of Black and Hispanic respondents to the right, educational differences have grown dramatically. By 2024, these educational differences were larger than racial differences. The growth of these educational differences has occurred not just in general, but also within racial groups, albeit to varying degrees.

## Results

I analyze the cooperative election study (CES) which includes surveys in each of the five presidential election years from 2008 to 2024, each with extremely large samples and overlapping sets of policy questions (7–11). I rely on a total of 132 policy items on topics including taxes, abortion, immigration, health care, and the environment in order to create measures of the ideology of the more than 250,000 respondents across these survey waves. These large samples allow for precise estimation, typically with at least several thousand observations for demographic subgroups within each year. Questions asked across multiple years serve as bridge items to create a single scale that is comparable both within and across years. To allow for straightforward interpretation, the ideology estimates are standardized so that the pooled (across years) mean and SD are 0 and 1, respectively, and so that lower (higher) values represent more liberal (conservative) ideological positions. The Materials and methods section and Supplementary material contain more information about the questions used and the estimation of respondent ideology.

### Decreasing ideological differences by race

**Fig. 1.**
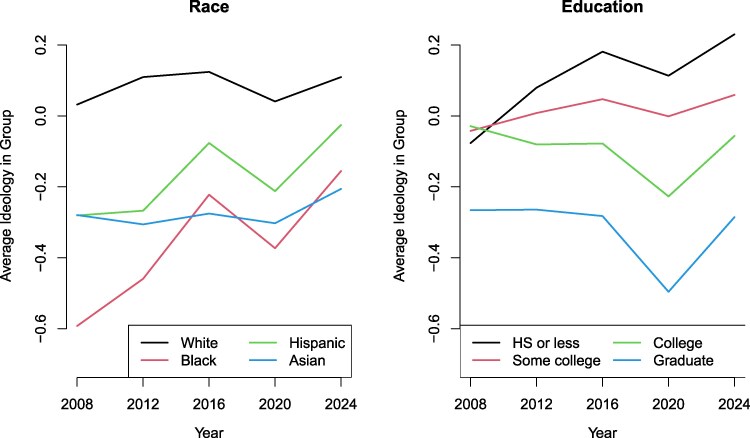
Average ideology estimates shown for respondents within each subgroup for each year of the CES survey. Left pane shows how racial differences in average ideology have decreased between 2008 and 2024. Right pane shows how educational differences in average ideology have widened over this same time.

The left pane of Figure 1 plots the average ideological position estimated for respondents in each racial group for each presidential election year (see Materials and methods section for more information about race codings). The most notable pattern is the dramatic shift in the ideology of the average Black respondent from 2008 to 2024. In 2008, Black respondents had an average ideology of −0.59 which put them well to the left of all other racial groups. In 2024, their average position was −0.16, a move of almost half a sample SD to the right, placing them as slightly more conservative than Asians, who are now the most liberal racial group on average.

Hispanic respondents also shifted to the right on average, moving just over one-fourth of a sample SD, while the average ideology of whites and of Asians showed only small fluctuations over this time period. The changes over this time period resulted in racial differences being much less stark in 2024 than they were in 2008. The gap between the average ideology of whites and Blacks shrank by nearly 60% over this time period, going from 0.62 to 0.27, while the difference between whites and Hispanics also decreased, albeit less dramatically. While racial differences were still present in 2024, they were much smaller in magnitude than they were less than two decades ago.

### Increasing ideological differences by education

Average ideological positions by education are plotted in the right pane of Figure 1. Putting aside people with graduate degrees, who have always been much more liberal than the rest of the public, ideology shows very small differences by education level in 2008. Respondents with high school degrees or less, those with some college, and those with college degrees all have average ideologies that are quite similar to one another. By 2024, the gap between these groups widened to over a quarter of a sample SD. The average ideological difference between those with a high school diploma or less and those with a college education is now larger than the difference between whites and Blacks on average, and if one considers Americans with graduate degrees, these educational differences are much larger in 2024 than are racial differences.

It is also notable that while much of the narrative about a potential educational realignment of American politics frames this change as being driven by Donald Trump (5), the growth of these educational differences appears to have begun in 2012 before Trump’s first full presidential campaign, although this trend continued to grow in subsequent years.

### Ideological differences by education have widened within each racial group

**Fig. 2.**
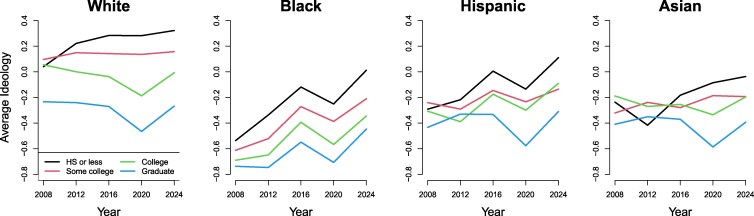
Trends in the average ideology of respondents by education level, plotted separately for each racial group. Panes from left to right show averages in each presidential election year between 2008 and 2024 among white, Black, Hispanic, and Asian respondents, respectively.

Figure 2 plots trends in the average ideology by level of education, separately shown for respondents within each racial category. For each race, educational differences have widened over this time period, albeit to different degrees. The most dramatic changes have been among whites, who in 2008 had very similar ideological positions whether they had college degrees or lower education levels (whites with graduate degrees were much more liberal). By 2024, these differences had widened considerably among whites, driven mostly by those with a high school degree or less becoming more conservative. Black respondents also showed some educational polarization with educational gaps widening over time, but all education subgroups among Blacks trended mostly in the conservative direction over this time. Hispanic and Asian respondents show somewhat similar dynamics, although the trends are slightly less clear and consistent. Overall, the ideological sorting by education seems to have occurred to a decent degree among all of these racial groups.

## Discussion

The results above demonstrate that over the past five presidential election years, ideological differences by race have declined dramatically, while education has become an increasingly prominent ideological divider. Americans’ ideologies have become more similar across races, driven primarily by Blacks moving sharply to the right, but also by Hispanics becoming slightly more conservative. Educational differences have increased among all racial groups, albeit to somewhat different degrees. These findings stand in contrast to others that have found that educational polarization in partisan vote margins, as opposed to the ideological positions studied here, has increased only among whites and not among minorities (2) and demonstrate that American politics have realigned not just in terms of previously documented partisan vote shares but also in terms of policy-based ideological positions.

### CES data

The CES data include the 2008 (n=32,800), 2012 (n=54,535), 2016 (n=64,600), 2020 (n=61,000), and 2024 (n=60,000) waves of the survey, each of which was fielded over the Internet by the company YouGov using their sample matching methodology to obtain representative samples from their large, nonrandomly selected respondent pool.

Respondent race was coded according to responses to the question “What racial or ethnic group best describes you?,” with those subsequently answering “Yes” to the question “Are you of Spanish, Latino, or Hispanic origin or descent?” also considered as Hispanic. For example, a respondent who initially says “Black,” but then identifies as Hispanic is coded as Hispanic (basing race only on responses to the first question results in extremely similar conclusions). Because respondents answering “Other” to the first race question were from a mix of many different races, non-Hispanic respondents from this category are not included in analyses involving race. Education was coded based on responses to the question “What is the highest level of education you have completed?” All binary (or easily binarized) questions on objective policies were used to measure ideology, but general questions or feelings toward specific political actors were not included. When estimating population quantities (eg subgroup means), weights provided with the survey were used. Full question wordings are included in the Supplementary material.

### Estimation of respondent ideology

A total of 132 unique policy items were used to measure respondent ideology, with some questions shared across multiple waves. These shared items are assumed to have the same item parameters (see below) in each wave they are asked, allowing for the estimation of respondent ideological positions on a common scale across years. Results are very similar when estimating ideology separately in each wave (year) of the survey data. The full list of questions, which is shown in the Supplementary material, includes a mix of items across social, economic, and other issue areas.

To estimate respondent ideology, a two parameter probit-link ideal point model (12, 13) is used $P(y_{ij}=1)=\Phi (\beta_{j}x_{i}-\alpha_{j})$, where $x_{i}$ is respondent *i*’s ideological position, $\alpha_{j}$ and $\beta_{j}$ are question-specific item parameters, and $y_{ij}$ is respondent *i*’s response on question *j*. The model’s parameters are estimated using the MCMCirt1d function in the MCMCpack package in R (14) Ideology estimates are standardized so that they have mean 0 and variance 1 across all respondents in all waves, with lower (higher) values corresponding to more liberal (conservative) ideological positions. Further details about the model’s priors and estimation are included in the Supplementary material.

## Supplementary Material

pgag102_Supplementary_Data

## Data Availability

Data are publicly available from CES website (see links in references). Code to reproduce all results can be found at https://doi.org/10.7910/DVN/CPDTXJ.
