# Supplementary material for: Americans’ ideological differences have decreased by race, increased by education
Source: PNAS Nexus. 2026 Apr 3;5(4):pgag102. doi: 10.1093/pnasnexus/pgag102 (PMC13071580; doi:10.1093/pnasnexus/pgag102)
Supplement: pgag102_Supplementary_Data [file pgag102_supplementary_data.pdf]

Supplementary Information for: Americans' Ideological Differences have Decreased by Race, Increased by Education PNAS-Nexus, 2026.

Stephen Jessee
sjessee@utexas.edu

March 31, 2026

Survey Questions Measuring Policy Views

Below is a list of the survey questions that are used as policy items (Y_{ij} in the statistical model's notation) to estimate respondents' ideological positions. Each item is listed along with the variable name for each wave in which the question appeared.

Abortion (2008:CC310, 2012:CC324) "Which one of the opinions on this page best agrees with your view on abortion? [By law, abortion should never be permitted; The law should permit abortion only in case of rape, incest or when the woman's life is in danger; The law should permit abortion for reasons other than rape, incest, or danger to the woman's life, but only after the need for the abortion has been clearly established; By law, a woman should always be able to obtain an abortion as a matter of personal choice]" (Note: coded as 1 if respondent answers "By law, a woman should always be able to obtain an abortion as a matter of personal choice") CC312 Privatize Social Security A proposal has been made that would allow people to put a portion of their Social Security payroll taxes into personal retirement accounts that would be invested in private stocks and bonds. Do you favor or oppose this idea? (2008: CC312)

Affirmative action programs give preference to racial minorities and to women in employment and college admissions in order to correct for discrimination. Do you support or oppose affirmative action? (2008: CC313; 2012: CC327)

Withdraw Troops Withdraw Troops from Iraq within 180 days (2008: CC316a)

Increase Minimum Wage from $5.15 to $7.25 (2008: CC316b)

Allow federal funding of embryonic stem cell research (2008: CC316c)

Allow U. S. spy agencies to eavesdrop on overseas terrorist suspects without first getting a court order (2008: CC316d)

Fund a $20 billion program to provide health insurance for children in families earning less that $43,000 (2008: CC316e)

Constitutional Amendment banning Gay Marriage (2008: CC316f)

Federal assistance for homeowners facing foreclosure and large lending institutions at risk of failing (2008: CC316g)

Extend the North American Free trade Agreement (NAFTA) to include Peru and Columbia (2008: CC316h)

U. S. Government's $700 Billion Bank Bailout Plan (2008: CC316i)

Do you favor or oppose the U. S. government guaranteeing health insurance for all citizens, even if it means raising taxes? (2008: CC417)

What do you think of a fuel tax to reduce greenhouse gas emissions? A gasoline tax of 25 cents per gallon and an electricity tax of $25 per month would reduce greenhouse gas emissions by approximately 40 percent

over the next 10 years. Would you support or oppose such a tax? (2008: CC422)

Grant legal status to all illegal immigrants who have held jobs and paid taxes for at least 3 years, and not been convicted of any felony crimes. (2012: CC322_1; 2016: CC16_331_1; 2020: CC20_331a; 2024: CC24_323a)

Increase the number of border patrols on the US-Mexican border. (2012: CC322_2; 2016: CC16_331_2; 2020: CC20_331b; 2024: CC24_323b)

Allow police to question anyone they think may be in the country illegally. (2012: CC322_3)

Fine US businesses that hire illegal immigrants. (2012: CC322_4)

Prohibit illegal immigrants from using emergency hospital care and public schools. (2012: CC322_5)

Deny automatic citizenship to American-born children of illegal immigrants. (2012: CC322_6)

Do you favor or oppose allowing gays and lesbians to marry legally? (2012: CC326; 2016: CC16_335)

2011 House Budget Plan. The Budget plan would cut Medicare and Medicaid by 42%. Would reduce debt by 16% by 2020 (2012: CC332A)

Simpson-Bowles Budget Plan. Plan would make 15% cuts across the board in Social Security, Medicare, Medicaid, and Defense, as well as other programs. Eliminate many tax breaks for individuals and corporations. Would reduce debt by 21% by 2020. (2012: CC332B)

The Middle Class Tax Cut Act. Would extend Bush era tax cuts for incomes below $200,000. Would increase the budget deficit by an estimated $250 billion. (2012: CC332C)

The Tax Hike Prevention Act. Would extend Bush-era tax cuts for all indviduals, regardless of income. Would increase the budget deficit by an estimated $405 billion. (2012: CC332D)

Birth Control Exemption. A Bill to let employers and insurers refuse to cover birth control and other health services that violate their religious beliefs. (2012: CC332E)

U.S.-Korea Free Trade Agreement. Would remove tariffs on imports and exports between South Korea and the U.S. (2012: CC332F)

Repeal Affordable Care Act. Would repeal the Affordable Care Act. (2012: CC332G; 2016: CC16_351I; 2020: CC20_327d; 2024: CC24_328d)

Keystone Pipeline. A bill to approve the Keystone XL pipeline from Montana to Texas and provide for environmental protection and government oversight. (2012: CC332G)

Affordable Care Act of 2010. Requires all Americans to obtain health insurance. Allows people to keep current provider. Sets up health insurance option for those without coverage. Increases taxes on those making more than $280,000 a year. (2012: CC332I)

End Don't Ask, Don't Tell. Would allow gays to serve openly in the armed services. (2012: CC332J)

Background checks for all sales, including at gun shows and over the Internet (2016: CC16_330a; 2024: CC24_321c)

Prohibit state and local governments from publishing the names and addresses of all gun owners (2016: CC16_330b; 2020: CC20_330a)

Ban assault rifles (2016: CC16_330d; 2020: CC20_330b; 2024: CC24_321a)

Make it easier for people to obtain concealed-carry permit (2016: CC16_330e; 2020: CC20_330c; 2024: CC24_321b)

Grant legal status to people who were brought to the US illegally as children, but who have graduated from a U.S. high school (2016: CC16_331_3)

Identify and deport illegal immigrants (2016: CC16_331_7)

Always allow a woman to obtain an abortion as a matter of choice (2016: CC16_332a; 2020: CC20_332a; 2024: CC24_324a)

Permit abortion only in case of rape, incest or when the woman's life is in danger (2016: CC16_332b; 2020: CC20_332b; 2024: CC24_324b)

Prohibit all abortions after the 20th week of pregnancy (2016: CC16_332c; 2020: CC20_332c)

Allow employers to decline coverage of abortions in insurance plans (2016: CC16_332d; 2020: CC20_332d)

Prohibit expenditure of funds authorized or appropriated by federal law for any abortion (2016: CC16_332e; 2020: CC20_332e)

Make abortions illegal in all circumstances (2016: CC16_332f; 2020: CC20_332f; 2024: CC24_324c)

Give Environmental Protection Agency power to regulate Carbon Dioxide emissions (2016: CC16_333a; 2020: CC20_333a; 2024: CC24_326a)

Raise required fuel efficiency for the average automobile from 25 mpg to 35 mpg (2016: CC16_333b)

Require a minimum amount of renewable fuels (wind, solar, and hydroelectric) in the generation of electricity even if electricity prices increase somewhat (2016: CC16_333c; 2020: CC20_333b)

Strengthen enforcement of the Clean Air Act and Clean Water Act even if it costs US jobs (2016: CC16_333d; 2020: CC20_333c; 2024: CC24_326c)

Eliminate mandatory minimum sentences for non-violent drug offenders (2016: CC16_334a; 2020: CC20_334a)

Require police officers to wear body cameras that record all of their activities while on duty (2016: CC16_334b; 2020: CC20_334b)

Increase the number of police on the street by 10 percent, even if it means fewer funds for other public services (2016: CC16_334c; 2020: CC20_334c; 2024: CC24_321d)

Increase prison sentences for felons who have already committed two or more serious or violent crimes (2016: CC16_334d)

Trans-Pacific Partnership Act Free trade agreement among 12 Pacific nations (Australia, Brunei, Canada, Chile, Japan, Malaysia, Mexico, New Zealand, Peru, Singapore, and the US). (2016: CC16_351B)

Education Reform. Repeals the No Child Left Behind Act, which required testing of all students and penalized schools that fell below federal standards. Allows states to identify and improve poor performing schools. (2016: CC16_351E)

Highway and Transportation Funding Act. Authorizes $305 Billion to repair and expand highways, bridges, and transit over the next 5 years. (2016: CC16_351F)

Iran Sanctions Act Imposes new sanctions on Iran, if Iran does not agree to reduce its nuclear program by June 30. (2016: CC16_351G)

Accountability and Cost Reform Act. Shifts Medicare from fee-for-service to pay-for-performance. Ties Medicare payments to doctors to quality of care measures. Requires higher premiums for seniors who make more than $134,000. Renews the Children Health Insurance Program (CHIP). (2016: CC16_351H)

Minimum wage. Raises the minimum wage to $12 an hour by 2020. (2016: CC16_351K)

Expand Medicare to a single comprehensive public health care coverage program that would cover all Americans. (2020: CC20_327a)

Allow the government to negotiate with drug companies to get a lower price on prescription drugs that would apply to both Medicare and private insurance. Maximum negotiated price could not exceed 120% of the average prices in 6 other countries. (2020: CC20_327b)

Lower the eligibility age for Medicare from 65 to 50 (2020: CC20_327c)

Restore the Affordable Care Act's mandate that all individuals be required to purchase health insurance. (2020: CC20_327e)

Allow states to import prescription drugs from other countries (2020: CC20_327f)

Withhold federal funds from any local police department that does not report to the federal government anyone they identify as an illegal immigrant. (2020: CC20_331c)

Reduce legal immigration by 50 percent over the next 10 years by eliminating the visa lottery and ending family-based migration. (2020: CC20_331d)

Increase spending on border security by $25 billion, including building a wall between the U.S. and Mexico (2020: CC20_331e)

Prohibit states from requiring that abortions be performed only at hospitals (not clinics). (2020: CC20_332g)

Raise the average fuel efficiency for all cars and trucks in the US from 40 miles per gallon to 54.5 miles per gallon by 2025 (2020: CC20_333d)

Decrease the number of police on the street by 10 percent, and increase funding for other public services (2020: CC20_334d; 2024: CC24_321e)

Ban the use of choke holds by police (2020: CC20_334e)

Create a national registry of police who have been investigated for or disciplined for misconduct (2020: CC20_334f)

End the Department of Defense program that sends surplus military weapons and equipment to police departments (2020: CC20_334g)

Allow individuals or their families to sue a police officer for damages if the officer is found to have "recklessly disregarded" the individual's rights. (2020: CC20_334h)

Tariffs on $200 billion worth of goods imported from China (2020: CC20_338a)

25% tariffs on all imported steel and 10% on imported aluminum EXCEPT from Canada and Mexico (2020: CC20_338b)

25% tariffs on all imported steel and 10% on imported aluminum INCLUDING from Canada and Mexico (2020: CC20_338c)

Increase tariffs on European aircraft and agricultural products. (2020: CC20_338d)

Amend federal laws to prohibit discrimination on the basis of gender identity and sexual orientation. (2020: CC20_350a)

Raise the minimum wage to $15 an hour. (2020: CC20_350b)

Confirm Brett Kavanaugh to become a Justice of the Supreme Court of the United States (2020: CC20_350c)

Require equal pay for women and men who are doing similar jobs and have similar qualifications. (2020: CC20_350d)

Provide permanent resident status to children of immigrants who were brought to the United States by their parents (also known as Dreamers). Provide these immigrants a pathway to citizenship if they meet the citizenship requirements and commit no crimes. (2020: CC20_350e; 2024: CC24_323d)

Remove President Trump from office for abuse of power (2020: CC20_350f)

Remove President Trump from office for obstruction of Congress (2020: CC20_350g)

In March, the CARES Act proposed to spend $2 trillion in emergency and health care assistance for individuals, families, and businesses, including up to $1,200 per individual and $500 per child. (2020: CC20_351a)

In May, the HEROES ACT proposed to spend an additional $3 trillion, including $1 trillion for state and local governments and hospitals, spend $200 billion in hazard pay for essential workers, and give households an additional $1,200 to $6,000. (2020: CC20_351b)

Withdraw the United States from the Paris Climate Agreement (2020: CC20_355a)

Withdraw the United States from the Trans-Pacific Partnership trade agreement, a free trade agreement that included the U.S., Japan, China, Australia, New Zealand, Canada, Chile, others. (2020: CC20_355b)

Repeal the Clean Power Plant Rules (the Clean Power Plant rules would require power plants to cut greenhouse gas emissions by 32 percent by 2030). (2020: CC20_355c)

Ban Transgender People in the Military (2020: CC20_355d)

Require able-bodied adults 18 to 49 years of age who do not have dependents to have a job in order to receive food stamps. (2020: CC20_355e)

Do you support or oppose confirming Amy Coney Barrett to become a Justice of the Supreme Court of the United States. (2020: CC20_356)

Ukraine - Do not get involved (2024: CC24_308a_1)

Ukraine - Send food, medicine, and other humanitarian aid to Ukraine (2024: CC24_308a_2)

Ukraine - Impose economic sanctions on Russia (2024: CC24_308a_3)

Ukraine - Provide arms to Ukraine (2024: CC24_308a_4)

Ukraine - Send military support staff to Ukraine (non-combat) (2024: CC24_308a_5)

Ukraine - Send significant force to fight Russia (2024: CC24_308a_6)

Ukraine - Negotiate a peace accord between Russia and Ukraine (2024: CC24_308a_7)

Ukraine - Send aid to the affected communities after the conflict to help rebuild destroyed schools, hospitals, and roads (2024: CC24_308a_8)

Israel/Gaza - Do not get involved (2024: CC24_308b_1)

Israel/Gaza - Send food, medicine and other aid to the communities affected (2024: CC24_308b_2)

Israel/Gaza - Provide arms to Israel (2024: CC24_308b_3)

Israel/Gaza - Provide arms to Hamas (2024: CC24_308b_4)

Israel/Gaza - Send the US Navy and troops to the region to keep the hostility from escalating (2024: CC24_308b_5)

Israel/Gaza - Send military support staff (non-combat) to Israel (2024: CC24_308b_6)

Israel/Gaza - Send military support staff (non-combat) to Gaza (2024: CC24_308b_7)

Israel/Gaza - Negotiate a peace settlement (2024: CC24_308b_8)

Israel/Gaza - Send aid to the affected communities after the conflict (2024: CC24_308b_9)

Increase spending on mental health and school safety Allow police to confiscate guns from people deemed to be dangerous by a judge Prohibit people convicted of domestic violence from owning guns; enhance background checks on minors Increase (2024: CC24_321f)

Build a wall between the U.S. and Mexico (2024: CC24_323c)

Expand access to abortion, including making it more affordable, broadening the types of providers who can offer care, and protecting access to abortion clinics (2024: CC24_324d)

Require that at least 20 percent of electricity be generated with renewable sources such as wind, solar, or hydroelectric power (2024: CC24_326b)

Increase fossil fuel production in the U.S. (2024: CC24_326d)

Halt new oil and gas leases on federal land (2024: CC24_326e)

Prevent the government from banning gas stoves (2024: CC24_326f)

Relax local zoning laws in your state to allow for construction of more apartments and condos. (2024: CC24_328a)

Expand federal tax incentives to encourage developers to build homes for people who make less than half of the average income in your area (2024: CC24_328b)

Require able-bodied adults under 64 years of age who do not have dependents to have a job in order to receive Medicaid. (2024: CC24_328c)

Expand Medicaid to cover individuals making less than $25,000 and families making less than $40,000 a year. (2024: CC24_328e)

Forgive up to $20,000 of student loan debt for each person (2024: CC24_323f)

Prohibit government restrictions on the provision of, and access to, contraceptives (2024: CC24_340a)

Prohibit government restrictions on the provision of, and access to, abortion services (2024: CC24_340b)

Require that all federal agencies recognize same-sex marriages and interracial marriages (2024: CC24_340c)

Ban TikTok unless China sells it to a US company (2024: CC24_340d)

Renew the federal surveillance programs that were adopted after 9/11 which allow the government to search private electronic data without a search warrant (2024: CC24_340e)

Deny access to asylum for immigrants who cross the US-Mexico border illegally (2024: CC24_340f)

Extend the tax cuts enacted in 2017, which reduced individual and corporate income tax rates and limited deductions on mortgage interest and state and local taxes (2024: CC24_341a)

Raise the corporate income tax rate from 21 percent to 28 percent (2024: CC24_341b)

Allow tax rates on those earning $400,000 or more a year to rise to 35 percent (2024: CC24_341c)

Spend $150 billion a year for 8 years on construction and repair of roads and bridges, rail, public transit, airports, water systems, broadband internet, and electric grid (2024: CC24_341d)

The use of race in university admissions violates Equal Protection under the Constitution and is illegal. (2024: CC24_445a)

The United States Constitution does not protect a woman's right to have an abortion. Roe v. Wade is overruled, and individual states can make their own laws governing abortion, including outlawing abortion. (2024: CC24_445b)

Model Estimation and Identification

The statistical model used to estimate respondent ideology is a two parameter probit-link ideal point model [2, 1] is used $P(y_{ij} = 1) = \Phi(\beta_j x_i - \alpha_j)$ where y_{ij} is respondent i's position on policy j. The model's parameters are estimated using the `MCMCirt1d` function in the `MCMCpack` package in `R`. Policy items that appeared in multiple waves (years) of the survey had the corresponding item parameters restricted to be

identical across years. This allows for the estimation of a single ideological scale on which the estimated ideal points (x) are comparable both within and across years.

Starting values for respondent ideologies x_i are obtained based on the proportion of conservative responses they gave to policy questions (note that the liberal/conservative direction of policies is not imposed in the subsequent model estimation). Default prior distributions are used which are independent standard normal for each x_i, and independent normal with mean 0 and standard variance 4 for each α_j and β_j The sampler is run for 50,000 "burn in" iterations which are discarded, and then run for an additional 250,000 iterations with every 100th iteration stored. This leaves 2,500 samples from the joint posterior over the model's unknown parameters.

To identify the model and to aid in interpretation of estimates, respondent ideal points x_i are rescaled at each iteration to have mean 0, standard deviation 1, and so that lower (higher) values represent more liberal (conservative) ideological positions. The point estimates (posterior means) are then similarly standardized.

References

[1] Joshua Clinton, Simon Jackman, and Douglas Rivers. The statistical analysis of roll call data. *American Political Science Review*, 98(2):355–370, 2004.

[2] Andrew D Martin and Kevin M Quinn. Dynamic ideal point estimation via markov chain monte carlo for the us supreme court, 1953–1999. *Political analysis*, 10(2):134–153, 2002.
